# Walking for leisure among adults from three Brazilian cities and its association with perceived environment attributes and personal factors

**DOI:** 10.1186/1479-5868-8-111

**Published:** 2011-10-13

**Authors:** Grace AO Gomes, Rodrigo S Reis, Diana C Parra, Isabela Ribeiro, Adriano AF Hino, Pedro C Hallal, Deborah C Malta, Ross C Brownson

**Affiliations:** 1 Physical Education Departament, Bioscience Institute, Physical Activity, Health and Sport Laboratory (NAFES), UNESP-Univ Estadual Paulista, Av. 24 A, 1515 Bela Vista, Rio Claro - SP, 13506-900, Brazil; 2 Physical Education Departament, CCBS, Pontiff Catholic University of Paraná, Rua Imaculada Conceição 1155, Curitiba - PR, 80215-901, Brazil; 3 Prevention Research Center in St. Louis, George Warren Brown School of Social Work. Washington University in St. Louis, 660 S. Euclid Avenue, St. Louis - MO, 63110, USA; 4 Epidemiology of Physical Activity Research Group, Federal University of Pelotas, Rua Marechal Deodoro 1160, Pelotas-RS, 96020-220, Brazil; 5 Health Surveillance Secretariat, Ministry of Health, Brasília-DF, Brazil; 6 Division of Public Health Sciences and Alvin J. Siteman Cancer Center, School of Medicine, Washington University in St. Louis, St. Louis-MO, 63110, USA

## Abstract

**Background:**

Walking is a popular form of physical activity and a convenient option to prevent chronic diseases. However, most of the evidence on this topic derives from high-income countries and little is known about walking patterns and its association with environmental features in low and middle income countries.

**Objectives:**

To describe walking for leisure and to identify its association with perceived environment and personal factors among residents of three state capitals from different regions of Brazil

**Methods:**

Cross sectional phone surveys were conducted in Recife, Curitiba and Vitória (n = 6,166) in 2007, 2008 and 2009 respectively. Physical activity was measured using the leisure-time sections of the long version of the International Physical Activity Questionnaire (IPAQ). Perceived environment characteristics were assessed using a modified version of the Neighborhood Environment Walkability Scale (NEWS). Multivariable analysis tested the associations between walking for leisure and perceived environment characteristics across the cities using logistic regression.

**Results:**

The proportions of respondents meeting physical activity recommendations through walking for leisure were 9.6%, 16.0% and 8.8% in Curitiba, Recife and Vitoria, respectively. Engaging in 150 min/wk or more of walking for leisure was significantly associated with younger age, higher education, better self-rated health and with lack of sidewalks on nearby streets. We did not find positive associations between walking for leisure and traffic conditions and safety related to cycling/walking during the day or night.

**Conclusion:**

Most environmental features were not associated with walking for leisure. Personal factors were stronger predictors of walking for leisure as compared with perceived environment factors.

## Introduction

Regular practice of physical activity is associated with reduced risk of developing chronic diseases and mortality [1-3]. In spite of the evidence about the benefits of physical activity for health, inactivity prevails in both high and low and middle income countries [4].

In high income countries, such as the United States, the percentage of people not meeting recommended levels of total physical activity is about 50,0% [5]. In addition, only 34,0% of people in the United States reports walking regularly [6]. Lack of physical activity is also a concern in low and middle income countries, such as Brazil. Studies have shown that only 10,5% to 21,5% % of people meet recommended levels for physical activity during leisure-time in several states from Brazil [7,8].

Physical inactivity is a complex behavior, determined by a series of factors at different levels. Over the last years, physical activity has been linked to personal barriers and to environmental factors [9,10]. The World Health Organization [4] cites some examples of environmental factors related to physical activity such as over-crowding, increased poverty, increased levels of crime, high levels of traffic, low air quality and lack of parks, sidewalks and sports and recreation facilities.

Changes in the environment can encourage people to be more physically active [11] and many environmental variables, such as accessibility or safety are significantly associated with physical activity [12]. Public health recommendations have emphasized common daily activities, such as climbing stairs, walking or bicycling to increase physical activity [13]. Walking is a popular form of physical activity and it has been described as a convenient and accessible option to promote health [14]. Additionally, walking has been shown as the most accessible way for achieving physical activity goals among groups who are typically sedentary, such as the elderly and low-income individuals [14,15].

There are few studies of the associations of the perceived environment and walking in Brazil [16,17]. Most studies have analyzed only the relationship with personal factors [18]. Also, most of the evidence on the influence of the perceived environment on physical activity is derived from high-income nations [12] and social, cultural and environmental factors in countries from Latin America such as Brazil vary greatly from those found in developed nations. The aims of the present study are: to describe the prevalence of walking for leisure in three state capitals from different regions of Brazil and to explore the association between walking for leisure and perceived environment and personal characteristics.

## Methods

### Study Settings

The state capitals of Recife, Curitiba and Vitória have different social and environmental characteristics; however, they have in common the fact that they provide public PA programs free of cost to their population, Academia da Cidade in Recife, CuritibAtiva in Curitiba and Serviço de Orientação ao Exercício (Exercise Orientation Service) in Vitoria [19-21]. The surveys from Recife and Curitiba were part of a larger effort implemented by Project GUIA (Guide for Useful Interventions for Physical Activity in Brazil and Latin America)[22,23] to better understand physical activity promotion in cities from Brazil. Table 1 shows some characteristics and indicators of the three cities related to population, traffic conditions and safety. Characteristics related to safety were included to describe the cities, population, automobile Fleet (units), inhabitants/cars and crime. The number of inhabitants/car can indicate less traffic density in the city. Curitiba has the smaller inhabitants/car ratio (2.1) indicating higher traffic density while Recife has a less dense traffic. Moreover, number of homicides by inhabitants is related with safety perception. In this sense Recife has a higher crime rate indicating a less safe environment while Curitiba is potentially safer compared to its counterparts.

**Table 1:** Sample characteristics in Recife, Curitiba and Vitória, Brazil, 2007-2009.

	Study site (year)	Recife (2007)	Curitiba (2008)	Vitória (2009)
**Sampling** **criteria**	Eligible respondents	3632	3406	2690
	Random sample	2400 households with at least 1 telephone landline from each stratum, 12 clusters of 200 telephone numbers each.	1000 people distributed across 9 strata and 1000 distributed in 4 extreme SES** strata.	Stratified according to presence or not of SOE* modules in the neighborhood
	Final sampling	2046	2097	2023
	Response rates	64,5%	60,5%	75,2%
**Environmental characteristics**	Population	1,561,659	1,851,215	320,156
	Automobile fleet (units)	307,166	867,066	109,305
	Inhabitants/cars	5.1	2.1	2.9
	Crimes (Homicides/100,000 inhabitants)	87.5	45.5	75.4

*SOE-Serviço de Orientação ao Exercício (Exercise Orientation Service)

** SES-Socio Economic Status

### Population and sample

Eligible respondents were non-institutionalized residents of the three cities who were 18 years or older. A random-digit-dialing telephone survey was applied using the methods of the Brazilian Chronic Disease Risk Factor Surveillance [7]. The coverage of land lines in Brazil is over 70% at the national level and we oversample low income populations since they tend to have lower access to telecommunications [24]. Stratified and clustered multistage sampling was used as detailed in Table 1. The sampling procedure was similar in all three cities with some differences in the stratification process which varied according to specific characteristics of the city. Institutional Review Board approval was obtained prior to data collection from São Paulo Federal University, Pontiff Catholic University of Parana in Curitiba and Washington University in St. Louis.

### Measures and data collection

A questionnaire was administered by trained interviewers with experience in telephone population surveys in 2007, 2008 and 2009. Averaging 20 minutes, the questionnaire included sociodemographic characteristics (gender, age, marital status, and education level); health (perceived health, self-reported weight and height); physical activity (walking for leisure-time); and perceived environment (accessibility and safety).

Body mass index (BMI) was calculated based on self-reported weight and height and was categorized as normal (less than 24.9 kg/m2), overweight (25-29.9 kg/m2) and obese (more than 30 kg/m2). The International Physical Activity Questionnaire (IPAQ) long version was used to assess physical activity. Walking for leisure was the dependent variable and a cutoff of 150 min/wk was used based on the most recent recommendations for physical activity and health [20].

Perceived environment information was obtained through a modified and culturally adapted version of the the Neighborhood Environment Walkability Scale (A-NEWS)[25] using categorical response options The modified version of the questionnaire was used in the three surveys. Prior studies with population from Brazil have shown that people have difficulty understanding questions in which the answer options are organized as a likert scale. Based on cognitive interviews during a pilot study and on prior research using the NEWs scale, several modifications to the response options as well as cultural adaptation to the questions and translation into Portuguese were done to the scale [26,27]. The modified scale has been previously used in other surveys in Brazil [16,28]. Only questions that were included in all three surveys were selected for this study to allow for comparability. These included perceptions of safety (walking/bicycling during the day and the night), traffic conditions, and presence of sidewalks.

### Data analysis

A descriptive analysis of walking for leisure according to personal and environmental factors, stratified by cities was conducted. A bivariate analysis was performed (using hierarchic model of logistic regression) between walking for leisure and selected independent variables stratifying by city. Three different models were run using multivariable logistic regression with walking for leisure as the dependent variable, stratifying by cities. We used the command svy to account for the complex sampling design and account for sampling weights. Model 1 included only demographic factors, model 2 included demographic factors, BMI, and perceived health, and model 3 included all previous variables plus perceived environment characteristics. We used the Stata 10 for data analysis.

## Results

### Study population characteristics

Table 2 shows the characteristics of the study population, which consisted of 2.276 men (41.2%) and 3.890 women (58.8%), with mean age of 45,0 (± 17,0). The education level varied across the three cities. In all three cities, the majority of the participants reported good health status (75.5%) and were married (48.0%). Overall, 59.7% were overweight by BMI (25-30 kg·m^2^), and the proportion of respondents that met physical activity recommendations through walking for leisure varied slightly between cities, 8.8%, 9.6% and 16.0% in Vitória, Curitiba and Recife, respectively. Most of the respondents reported presence of sidewalks on nearby streets (75.9%) and perceived safety when cycling/walking during the night (59.2%); however, cycling/walking during the day was not considered safe by the majority (80.6%) of the respondents in all three cities. More than half of the participants reported that traffic makes cycling/walking more difficult, this proportion was higher in Vitória (62.1%) than in Curitiba (54.9%) and Recife (43.6%).

**Table 2:** Demographic characteristics of participants according to the city of residence, Brazil, 2007-2009

Variables	Categories	Curitiba		Recife		Vitória		All	
		**n**	**%^1^**	**n**	**%^1^**	**n**	**%^1^**	**n**	**%^1^**
		
Gender	Men	768	37.4	761	43.7	747	37.8	2276	39.8
	Women	1,329	62.6	1,285	56.3	1,276	62.2	3890	60.2
Age categories	16-34	611	47	700	47.6	614	44.8	1925	35.1
	35-45	861	37.3	761	34.1	798	35	2420	39.7
	55+	625	15.6	585	18.3	611	20.2	1821	25,5
Education level	< High	671	28.6	631	46.1	492	20.4	1794	34.1
	High school	724	41.2	765	38.2	652	33.6	2141	34.7
	> High school	692	30.1	612	15.7	879	46.0	2183	31.2
Marital status	Single	522	34.7	764	46.3	603	38.7	1889	33.1
	Married	1,199	56	940	42.9	1053	50.4	3192	50.5
	Other	376	9.3	342	10.9	367	10.9	1085	16.4
Perceived health	Poor/Regular	541	24.6	774	37.8	608	27.7	1923	29.6
	Good	963	48.0	822	41.6	771	38.8	2556	38.7
	Very good/excellent	592	27.5	450	20.6	631	33.6	1673	31.8
Body mass index	Normal	1,133	60.2	1,115	58.1	1,010	56.7	3258	59.7
	Overweight/Obese	912	39.8	830	41.9	888	43.3	2630	40.3
Walking for leisure (150 min/week)	Yes	361	15.1	378	14.3	387	17.6	5032	14.7
	No	1,736	84.9	1,666	85.7	1,630	82.4	1126	85.3
Sidewalks on nearby streets	No	541	29.3	284	18.9	1,036	53.3	1861	24.2
	Yes	1,556	70.7	1762	81.1	936	46.7	4254	75.8
Traffic makes it difficult to cycle/walk	No	967	45.1	1,077	56.4	692	37.9	2736	51.2
	Yes	1,130	54.9	968	43.6	1,231	62.1	3329	48.8
Safe to cycle/walk during the night	No	1,760	84.8	1,551	79.5	1,128	58.2	4439	80.5
	Yes	337	15.2	495	20.5	816	41.8	1648	19.5
Safe to cycle/walk during the day	No	775	37.2	806	44.4	408	21.6	1989	40.5
	Yes	1,322	62.8	1,240	55.6	1,530	78.4	4092	59.5

^1^Weighed prevalence rates

### Individual and environmental correlates of walking for leisure

Results of crude and adjusted logistic regression are depicted in Tables 3 and 4, respectively. The associations found in the crude analysis remained even after adjusting for potential confounders. Logistic regression analysis showed that younger respondents (16-34 yrs) tended to walk for leisure more in all three cities ((Odds Ratio (OR) = 3.0, Confidence Interval (CI) = 2.1-4.3). With the exception of Curitiba, higher levels of education (OR = 1.9, CI = 1.4-2.6) and better self-rated health (OR = 1.8, CI = 1.3-2.4) were found to be associated with walking for leisure time. Walking for leisure was negatively associated with presence of sidewalks nearby in the city of Vitória. No statistical associations were found with sex, marital status and BMI in relation to walking for leisure time in any of the cities.

**Table 3:** Unadjusted prevalence odds ratios for personal and environmental factors associated with walking in leisure time, Brazil, 2007-2009.

Variables	Categories	Curitiba^1^		Recife^1^		Vitoria^1^		All^1^	
	
		%	OR (CI)	%	OR (CI)	%	OR (CI)	%	OR (CI)
Gender	Men	15,3	0.9 (0.7-1.3)	13,6	1.1 (0.7-1.5)	18,1	0.9 (0.7-1.2)	14,3	1.0 (0.8-1.3)
	Women	14,9	Ref	14,8	Ref	17,3	Ref	15,0	Ref
Age categories	16-34	13,1	1.8 (1.2-2.7)	12,3	2.3 (1.5-3.7)	11,8	2.6 (1.9-3.7)	11,0	2.1 (1.6-2.9)
	35-45	14,7	1.1 (0.7-1.6)	13,3	1.9 (1.2-3.0)	20,0	1.8 (1.3-2.5)	16,4	1.5(1.1-2.1)
	55+	22,0	Ref	21,8	Ref	26,3	Ref	21,2	Ref
Education level	< High	14,9	Ref	12,3	Ref	16,4	Ref	13,2	Ref
	High school	12,1	0.7 (0.5-1.1)	13,3	1.0 (0.7-1.6)	16,1	0.9 (0.6-1.3)	12,9	1.6 (1.2-2.2)
	> High school	19,5	1.3 (0.9-2.0)	21,8	1.9 (1.2-3.0)	19,3	1.2 (0.8-1.6)	20,4	0.9 (0.7-1.3)
Marital status	Single	13,9	1.5 (0.9-2.5)	10,5	2.8 (1.5-5.2)	15,1	1.5 (1.0-2.2)	11,8	2.2(1.5-3.4)
	Married	15,0	1.0 (0.7-1.5)	15,4	1.5(1.0-2.2)	18,8	1.3 (0.9-1.7)	15,4	1.3 (1.0-1.7)
	Other	20,0	Ref	25,3	Ref	21,2	Ref	23,4	Ref
Perceived health	Poor/Regular	13,7	Ref	13,1	Ref	14,4	Ref	13,3	Ref
	Good	12,8	0.9 (0.6-1.3)	13,0	0.9 (0.6-1.5)	17,8	1.2 (0.9-1.7)	13,1	0.9 (0.7-1.3)
	Very good/ excellent	20,2	1.5 (1.0-2.4)	19,1	1.5 (0.9-2.4)	20,2	1.5 (1.0-2.1)	19,7	1.5 (1.1-2.1)
Body mass index	Normal	15,9	0.9(0.6-1.2)	14,2	0.9 (0.6-1.3)	16,3	1.2(0.9-1.5)	14,9	0.9 (0.7-1.1)
	Overweight/ Obese	14,6	Ref	13,6	Ref	19,2	Ref	14,2	Ref
Sidewalks on t nearby streets	No	11,6	1.5 (1.0-2.2)	8,0	2.1(1.1-3.9)	15,7	1.3 (1.0-1.7)	10,3	1.6 (1.2-2.2)
	Yes	16,5	Ref	15,8	Ref	19,9	Ref	16,1	Ref
Traffic makes it difficult to cycle/walk	No	13,6	Ref	14,3	Ref	17,2	Ref	14,2	Ref
	Yes	16,8	0.7(0.5-1.0)	14,3	1.0 (0.7-1.4)	18,0	1.0 (0.8-1.4)	15,2	0.9 (0.7-1.1)
Safe to cycle/walk during the night	No	17,9	0.8 (0.6-1.0)	15,5	0.7 (0.5-0.9)	17,0	0.9 (0.6-1.2)	16,4	0.8 (0.630-1.021)
	Yes	13,4	Ref	13,3	Ref	18,4	Ref	13,6	Ref
Safe to cycle/walk during the day	No	15,4	0.8 (0.5-1.2)	14,2	1.0 (0.6-1.4)	19,1	0.8 (0.6-1.0)	14,8	0.9 (0.7-1.2)
	Yes	13,1	Ref	14,4	Ref	16,0	Ref	14,2	Ref

^1^Weighed prevalence rates and prevalence odds ratios

**Table 4:** Adjusted prevalence odds ratios for personal and environmental factors associated with walking in leisure time, Brazil, 2007-2009.

Variables	Model*	Categories	Curitiba		Recife		Vitoria		All	
	
			Adjusted OR^1^ (95% CI)	p-value	Adjusted OR^1^ (95% CI)	p-value	Adjusted OR^1^ (95% CI)	p-value	Adjusted OR^1^ (95% CI)	p-value
Gender	1	Men	Ref		Ref		Ref		Ref	
		Women	0.9 (0.7-1.3)	0.90	1.0 (0.7-1.5)	0.64	1.0 (0.7-1.2)	0.86	1.0 (0.8-1.2)	0.84
Age categories	1	16-34	2.0 (1.2-3.4)	**0.00**	4.3 (2.6-7.1)	**0.00**	4.2 (2.8-6.5)	**0.00**	3.0 (2.1-4.3)	**0.00**
		35-45	1.2 (0.8-1.9)	0.30	3.1 (1.9-5.0)	**0.00**	2.3 (1.6-3.4)	**0.00**	2.0 (1.4-2.7)	**0.00**
		55+	Ref		Ref		Ref		Ref	
Education level	1	< High	Ref		Ref		Ref		Ref	
		High school	1.5 (1.0-2.2)	**0.04**	1.5 (1.0-2.4)	0.03	1.3 (0.8-2.1)	0.15	1.3 (0.9-1.7)	0.07
		> High school	0.8 (0.5-1.3)	0.61	2.1 (1.3-3.3)	**0.00**	1.6 (1.0-2.5)	**0.02**	1.9 (1.4-2.6)	**0.00**
Marital status	1	Single	1.2 (0.6-2.1)	0.47	1.1 (0.6-2.1)	0.62	0.7 (0.5-1.0)	0.19	1.2 (0.8-1.8)	0.36
		Married	1.0 (0.6-1.5)	0.22	0.9 (0.6-1.5)	0.87	0.7 (0.4-1.1)	0.08	0.9 (0.7-1.3)	0.99
		Other	Ref		Ref		Ref		Ref	
Perceived health	2	Poor/Regular	Ref		Ref		Ref		Ref	
		Good	0.9 (0.6-1.4)	0.77	1.2 (0.8-1.8)	0.30	1.4 (0.9-2.1)	0.07	1.1 (0.8-1.4)	0.49
		Very good/excellent	1.5 (0.9-2.4)	**0.05**	2.2 (1.4-3.4)	**0.00**	1.7 (1.1-2.6)	**0.01**	1.8 (1.3-2.4)	**0.00**
Body mass index	2	Normal	0.8 (0.6-1.1)	0.35	0.8 (0.6-1.1)	0.35	1.1 (0.8-1.5)	0.25	0.8 (0.6-1.0)	0.22
		Overweight/Obese	Ref		Ref		Ref		Ref	
Sidewalks on nearby streets	3	No	1.2 (0.8-1.8)	0.34	1.8 (0.9-3.5)	0.08	1.3 (1.0-1.7)	**0.04**	1.5 (1.0-2.1)	**0.01**
		Yes	Ref		Ref		Ref		Ref	
Traffic makes it difficult to cycle/walk	3	No	Ref		Ref		Ref		Ref	
		Yes	0.8 (0.5-1.1)	0.22	1.0 (0.7-1.5)	0.63	0.9 (0.7-1.3)	0.88	0.9 (0.7-1.2)	0.77
Safe to cycle/walk during the night	3	No	0.7 (0.5-1.0)	0.09	0.8 (0.5-1.2)	0.42	0.9 (0.6-1.2)	0.61	0.8 (0.6-1.0)	0.12
		Yes	Ref		Ref		Ref		Ref	
Safe to cycle/walk during the day	3	No	0.9 (0.5-1.5)	0.83	0.9 (0.6-1.4)	0.87	0.8 (0.6-1.1)	0.23	0.9 (0.7-1.3)	0.93
		Yes	Ref		Ref		Ref		Ref	

^1^Weighed prevalence odds ratio adjusted for Gender, Age categories, Education level, Marital status, Perceived health and BMI; ^2^Weighed prevalence odds ratio adjusted for Gender, Age categories, Education level, Marital status, Perceived health, BMI and City

* Model: level 1 = demographics; level 2 = BMI and perceived health; level 3 = perceived environment variables

The adjusted logistic regression in the combined analysis (all three cities) showed some associations. Age group was significantly correlated with meeting recommendations through walking for leisure time. Younger age, having more than high school and reporting very good/excellent perceived health were found to be positively and significantly associated with walking for leisure. Presence of sidewalks on nearby streets was the only perceived environmental factor found to be associated with walking for leisure in a negative direction in the city of Vitoria.

## Discussion

This is one of the first studies examining personal and environmental factors associated with walking for leisure across cities in Brazil. We found that higher levels of walking for leisure were associated with lower age, higher educational status and better perceived health in all cities and with lack of nearby sidewalks in the city of Vitória and in the combined data. No associations were found with sex, marital status, BMI, perceived traffic and perceived safety to cycle/walk during day or night across all three cities. Some of the perceived environment characteristics presented correlations in the opposite directions than expected; for instance, presence of sidewalks was negatively associated with a higher likelihood of walking during leisure time.

Our findings can be interpreted in light of other research from the region. For example, Matsudo and colleagues [29] examined trends of physical activity during leisure time in different regions of Brazil from 2002 to 2008. Taking into account geographic region, people from the coastline were more active than the ones from the countryside and the ones from the metropolitan region. Similarly, Moura et al. [7] found the highest rates of leisure time physical activity in Vitória (21.2%) and the lowest in Recife (15.0%) out of all the cities from Brazil. Our data, which only looked at walking for leisure, found different rates, the lowest level of walking for leisure was 8.8% in Vitoria versus 16.0% in Recife, both coastal cities from the country. It is possible that the majority of the reported physical activity during leisure time in Vitoria and Recife in the Matsudo study corresponded to moderate and vigorous physical activity and not necessarily walking. Regarding personal characteristics, our findings are consistent with most of the national and international literature, in that, younger age, higher educational level, and better perceived health are shown to be positively associated with physical activity [8,18,30-32].

In addition, according to findings from all State capitals of Brazil, men tend to be more active during leisure time when compared to women [8,31,32]. In our study, the proportion of women that walk for leisure (15.0%) was higher than the proportion of men (14.3%); sex was not an effect modifier of the associations. Simões et al. [20] found that men were more active than women during leisure time in Recife, taking into account vigorous, moderate and walking during leisure, and not just walking like in this case. This could explain the differences found in this study which used the same database for Recife.

Research derived from high and low-middle income countries, shows associations between several perceived environment attributes and physical activity [16,33,34], and in particular with walking for leisure [35,36]. Duncan et al. [11] conducted a meta-analysis of studies examining the association between perceived environment and physical activity, they found that perceived environment has a modest, yet significant association with physical activity. In our study we did not find any correlations between perceived environment attributes with the exception of a negative correlation between having sidewalks on nearby streets and walking for leisure in the city of Vitoria. The same finding was observed in the combined model but it is probably explained in its entirety by the strong association found in Victoria. Our inability to find significant associations may be due to the fact that some of the characteristics of the environment captures with the scale used are not sensible for identifying critical features related to the culture and social environment factors. Further research should explore in more detail which are the characteristics and factors of the environment that are associated with practice of physical activity in Brazil. We indicated some environment differences about population, number of automobiles and crimes among the cities, however they were not able to explain the results. In addition, self reported information in regards to features of the environment are likely to differ from those captured with objective methods. Thus, the use of geographic information systems in studies that explore the association between the environment and physical activity levels is needed.

The contradictory finding of a positive association between walking for leisure and lack of sidewalks on nearby streets, could be explained by the fact that in some cities of Brazil sidewalks may serve more as a barrier rather that a facilitator for walking. This is due to their poor quality and maintenance as well as overcrowding which limits the ability and the enjoyment of walking. This highlights the importance of developing scales that are culturally relevant and context specific for cities in Latin America, that have very different characteristics from cities found in North America and Europe. Despite the cultural adaptation of the A-News scale conducted for this study, the scale is capturing attributes of the environment that are based on findings from studies conducted in the United States, which has significant differences in terms of socio-demographic, economic, and cultural characteristics when compared to Brazil [37].

This study adds to the evidence base on determinants of physical activity by incorporating a range of individual and environmental measures. It is one of the few such studies from Latin America. In summary, personal factors were more strongly related to walking for leisure than perceived environmental features. Further studies should explore other environmental characteristics, including similar analyses in other cities in Brazil and Latin America. Future research should also examine these associations longitudinally.

## List of abbreviation used

PA: physical activity.

## Competing interests

The authors declare that they have no competing interests.

## Authors' contributions

All authors made substantial contributions to the design of the study. GAOG analyzed and interpreted the data and wrote the draft version. RR and AAFH and RR were involved in the acquisition of the data. IR, DCP, DM, PH and RB were involved in the writing of the paper and critical revision of the manuscript, and have given their approval for the submitted manuscript. All authors read and approved the final manuscript.
